# Bone turnover: the role of lipoproteins in a population-based study

**DOI:** 10.1186/s12944-024-02290-y

**Published:** 2024-09-19

**Authors:** Todd Winckel, Nele Friedrich, Stephanie Zylla, Marc Fenzlaff, Juliane Schöpfel, Karen Friederike Gauß, Astrid Petersmann, Matthias Nauck, Henry Völzke, Anke Hannemann

**Affiliations:** 1 Institute of Clinical Chemistry and Laboratory Medicine, University Medicine Greifswald, Ferdinand-Sauerbruch-Straße, D-17475 Greifswald, Germany; 2 DZHK (German Centre for Cardiovascular Research), Partner Site Greifswald, Greifswald, Germany; 3 Institute for Clinical Chemistry and Laboratory Medicine, University Medicine Oldenburg, Oldenburg, Germany; 4 Institute for Community Medicine, University Medicine Greifswald, Greifswald, Germany

**Keywords:** Bone turnover, Bone stiffness index, Lipid metabolism, Lipoprotein subclasses, General population, Cross sectional analyses

## Abstract

**Background:**

Dyslipidemia has been associated with reduced bone mineral density and osteoporotic fractures, but the relation between lipid and bone metabolism remains poorly understood. Analysing the effects of lipoprotein subclasses on bone turnover may provide valuable insights into this association. We therefore examined whether lipoprotein subclasses, measured by proton nuclear magnetic resonance (^1^H-NMR) spectroscopy, are associated with bone turnover markers (BTMs) and with the ultrasound-based bone stiffness index.

**Methods:**

Data from 1.349 men and 1.123 women, who participated in the population-based Study of Health in Pomerania-TREND were analysed. Serum intact amino-terminal propeptide of type I procollagen (P1NP, bone formation) and carboxy-terminal telopeptide of type I collagen (CTX, bone resorption) concentrations were measured. Associations between the lipoprotein data and the BTMs or the stiffness index were investigated using linear regression models.

**Results:**

The triglyceride or cholesterol content in very-low-density lipoprotein and intermediate-density lipoprotein particles was inversely associated with both BTMs, with effect estimates being slightly higher for CTX than for P1NP. The triglyceride content in low-density lipoprotein and high-density lipoprotein particles and the Apo-A2 content in high-density lipoprotein particles was further inversely associated with the BTMs. Associations with the ultrasound-based bone stiffness index were absent.

**Conclusions:**

Consistent inverse associations of triglycerides with bone turnover were observed, which argue for a protective effect on bone health, at least in the normal range. Yet, the presented associations did not translate into effects on the ultrasound-based bone stiffness. Further, there was no relevant gain of information by assessing the lipoprotein subclasses. Nevertheless, our study highlights the close relations between lipid and bone metabolism in the general population.

**Supplementary Information:**

The online version contains supplementary material available at 10.1186/s12944-024-02290-y.

## Background

Unfavourable alterations in the human blood lipid profile [1] as observed in dyslipidemia, represent a cornerstone in the development of atherosclerosis and cardiovascular disease. Dyslipidemic alterations include elevations of low-density lipoprotein (LDL)-cholesterol concentrations and decreases of high-density lipoprotein (HDL)-cholesterol concentrations. While elevations in LDL-cholesterol concentrations have been linked to endothelial dysfunction and vascular inflammation [2], decreases in HDL-cholesterol concentrations promote cardiovascular disease, as it exerts cardio- and atheroprotective effects [3]. Also elevated lipoprotein(a) (Lp(a)) concentrations play a fundamental causal role in the development of atherosclerotic disease [4]. Apart from its adverse cardiovascular effect, dyslipidemia relates to severe diseases in other organ systems [1]. In fact, several epidemiological studies suggested associations with low bone mineral density (BMD) or fractures (for a review see [5]). These associations may explain the co-occurrance of osteoporosis and atherosclerosis in dyslipidemic patients [6]. Proposed pathophysiological mechanisms underlying this relation include interference of low HDL-cholesterol concentrations with osteoblastic function and differentiation [7] and consequently a reduction of bone mass. Another pathophysiological link involves sclerostin, a key antagonist of the Wnt signalling pathway. In dyslipidemia, an excessive secretion of sclerostin is triggered, which results in inhibition of Wnt-mediated osteoblast activation and bone formation (reviewed in [8]). Further, bone marrow adiposity is linked to processes that favour adipoblastic over osteoblastic differentiation [7].

As osteoporosis is of high importance in the aging population, leading to a diminished quality of life and high morbidity, its pathophysiology needs to be better understood. For this, a more detailed insight into the relationship between lipid and bone metabolism is needed and may be achieved by analyzing lipoprotein subclasses. Lipoproteins are complex particles that transport cholesterol and triglycerides in the circulation. They differ in size, density, composition and function [9]. For the determination of cardiovascular risk, cholesterol concentrations in LDL and HDL particles, triglycerides and, once in a lifetime, Lp(a) are evaluated in clinical practice [10]. Further lipoproteins, including chylomicrons, chylomicron remnants, very-low density lipoprotein (VLDL) and intermediate density lipoprotein (IDL), are currently not assessed in patient care. Also subclasses of VLDL, LDL and HDL particles are only evaluated for research purposes. Yet, these subclasses have been found to be of relevance for cardiovascular health [9]. Thus, small and dense LDL particles are more atherogenic than large, less dense LDL particles [11, 12], while small and dense HDL particles are less atheroprotective than large, less dense HDL particles [13]. Also the composition of the LDL particles, i.e. the content of cholesterol and triglycerides, is of relevance for cardiovascular risk. The LDL triglyceride content is for example related to atherosclerotic phenotypes [14]. Whether similar observations, i.e. diverging effects depending on the lipoprotein subclasses, are also present in bone metabolism is yet unknown. Respective studies may generate hypotheses on how changes in the lipid profile affect bone metabolism but research in this area is lacking. Therefore, we aimed to analyze whether and how various lipoprotein subclasses, measured via proton nuclear magnetic resonance spectroscopy (^1^H-NMR), are associated with bone formation or resorption processes and with the ultrasound-based bone stiffness index in adult men and women.

## Methods

### The Study of Health in Pomerania-TREND (SHIP-TREND)

SHIP-TREND is a population-based cohort study that includes adult men and women [15]. The study population is based on a representative sample of the 20–79 year old inhabitants of the study region in Northeast Germany. Baseline examinations in SHIP-TREND were performed between 2008 and 2012 including 4,420 participants (response 50.1%). Further details on study design, sampling procedures and rationale are given elsewhere [15].

### Study population

**Fig. 1:**
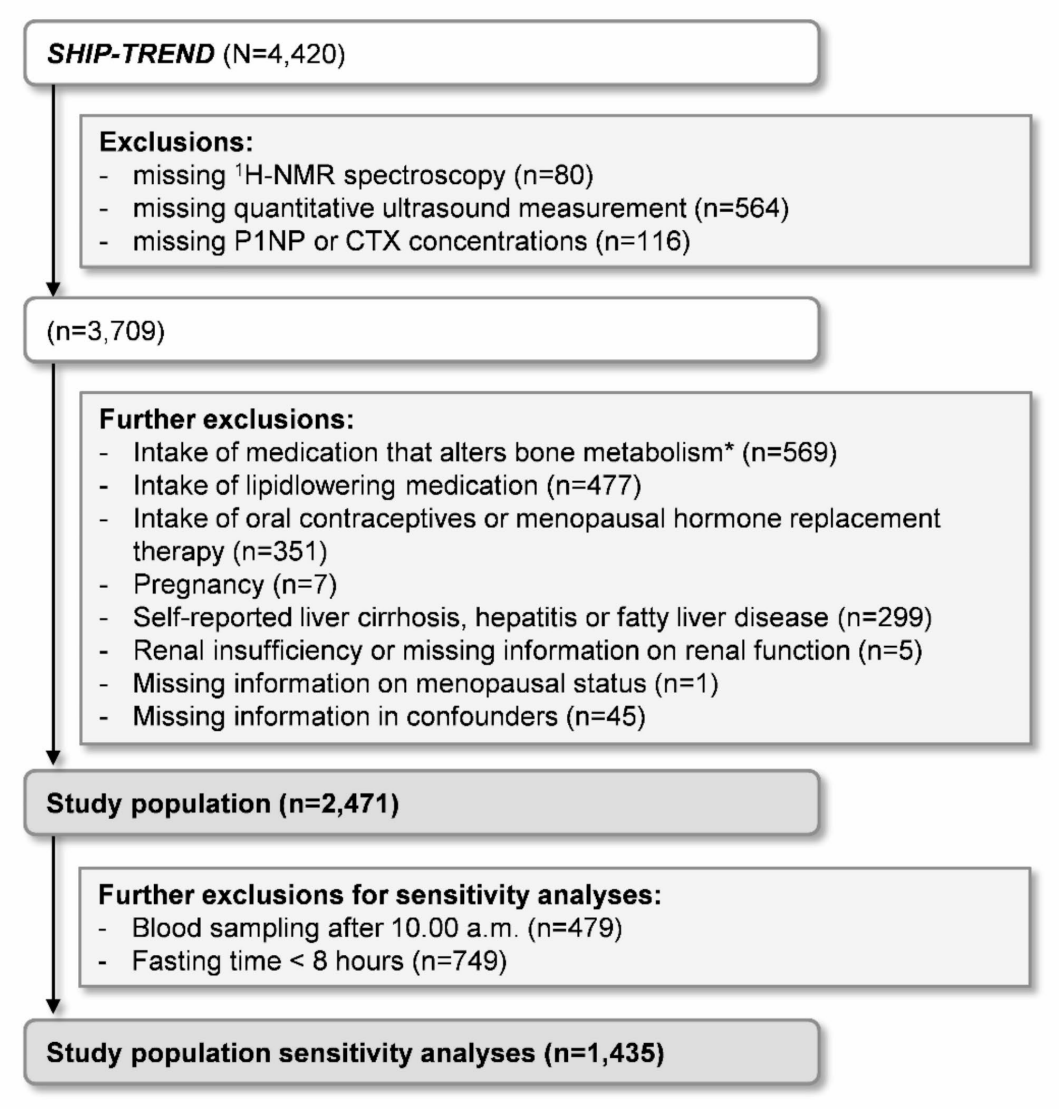
Selection of the study population. *systemic glucocorticoids, antiepileptics, aromatase inhibitors, antidepressants, bisphosphonates, selective estrogen receptor modulators and parathyroid hormone. CTX, carboxy-terminal telopeptide of type I collagen; ^1^H-NMR, proton nuclear magnetic resonance spectroscopy; P1NP, intact amino-terminal propeptide of type I procollagen; SERM, selective estrogen receptor modulators; SHIP-TREND, Study of Health in Pomerania-Trend

For the present cross-sectional analyses, all SHIP-TREND participants without ^1^H-NMR measurement and quantification of lipoprotein subclasses, with missing quantitative ultrasound data or without measurement of bone turnover markers (BTM) were excluded from the study population (Fig. 1). We then excluded certain diseases and conditions, that highly impact on lipid or bone metabolism. This comprised intake of systemic glucocorticoids, antiosteoporotic drugs, antiepileptics, aromatase inhibitors and antidepressants, intake of lipidlowering medication, oral contraceptives or menopausal hormone replacement therapy, pregnancy, self-reported liver cirrhosis, hepatitis or fatty liver disease and renal insufficiency or missing information on renal function. Finally, all women with missing information on their menopausal status and all participants with missing information in any of the confounders were excluded. This resulted in a study population of 2,471 participants. In a sensitivity analysis, all non-fasting participants (fasting for less than eight hours) and all participants whose blood samples were taken after 10.00 a.m. were excluded, resulting in a population of 1,435 participants.

### Measurements

The SHIP-TREND program offered a broad range of medical examinations to the study participants. These included, for example, a computer-assisted personal interview on lifestyle and medical history, a quantitative ultrasound measurement at the heel and blood sampling.

During the personal interview, participants were asked for their physical activity and those who reported less than one hour of regular activity during summer and winter were classified as physically inactive. Participants were further asked for the presence of liver disease, diabetes mellitus and for their regular medication intake. Information on the intake of glucocorticoids, antiosteoporotic drugs including bisphosphonates, selective estrogen receptor modulators and parathyroid hormone, as well as lipid lowering medication, oral contraceptives and menopausal hormone therapy were documented. All women aged 60 years or older and all women between 40 and 60 years of age without self-reported regular menstrual cycling were considered postmenopausal, all remaining women as premenopausal. Waist circumference was measured and venous blood samples were taken. The blood samples were taken in the mornings and information on fasting status and the exact blood sampling time were documented. Serum and plasma samples were stored at -80 °C in the Integrated Research Biobank (Liconic, Lichtenstein) of the University Medicine Greifswald. Serum intact amino-terminal propeptide of type 1 procollagen (P1NP) and C-terminal telopeptides of type 1 collagen (CTX) concentrations were determined by automated chemiluminescent immunoassays on the IDSiSYS Multi-Discipline Automated Analyser (Immunodiagnostic Systems Limited, Frankfurt am Main, Germany). The estimated glomerular filtration rate was calculated from serum creatinine according to the four-variable Modification of Diet in Renal Disease formula. Renal insufficiency was defined as values < 30 ml/min/1.73 m². More details on the measurements are given in the additional material.

### Quantitative ultrasound measurements

Quantitative ultrasound measurements were performed by trained and certified examiners. The measurements were performed using the water-based bone ultrasonometer Achilles InSight (GE Medical Systems Ultrasound, GE Healthcare, USA). It measures the frequency-dependent attenuation and the speed of the sound waves that pass through an individual’s heel (os calcis). Both characteristics are combined to form the stiffness index according to the following formula: stiffness index = (0.67 × broadband ultrasound attenuation) + (0.28 × speed of sound) − 420. Higher stiffness index values indicate better bone properties. Stiffness indices below the mean − 2.5 standard deviations (SD) in a young healthy reference population indicate a high osteoporotic fracture risk, indices between − 1 and − 2.5 SD indicate a medium risk and indices above − 1 SD indicate a low osteoporotic fracture risk. The measurements were performed successively on both feet and the data from the foot with the lower stiffness index was used for statistical analyses.

### Laboratory methods

#### ^1^H-NMR measurements and quantification of lipoprotein subclasses

A detailed description of the quantification of the lipoprotein subclasses and their correlation is given in the additional methods. Briefly, spectra were recorded on one of three Bruker AVANCE-II 600 NMR spectrometer operated by TOPSPIN 3.2 software (both Bruker Biospin, Rheinstetten, Germany), equipped with 5-mm z-gradient probe (Bruker Biospin, Rheinstetten, Germany) and automated tuning and matching unit (Bruker Biospin, Rheinstetten, Germany). In cooperation with the Institute of Clinical Chemistry and Laboratory Medicine at the University Medicine Greifswald, Bruker developed an automatic analysis tool to quantify lipoprotein subclasses from NMR spectra. In the framework of this process the lipoprotein measurements were performed by ultracentrifugation, the gold standard method to analyse lipoproteins [16], in Greifswald. Based on this development, the spectra of the present study were submitted to data analysis for lipoprotein subclass and apolipoprotein analysis B.I.LISA™ (Bruker BioSpin GmbH Germany).

For the present study, data on the cholesterol, phospholipid and triglyceride content in VLDL (total and subclasses 1–5), IDL, LDL (total and subclasses 1–6) and HDL (total and subclasses 1–4) particles were examined. Futhermore, information on ApoA1 and ApoA2 protein content in HDL-particles (total and subclasses 1–4), as well as information on ApoB-100 protein content in total VLDL, total IDL, total LDL and LDL 1–6 particles was analysed. This yielded a total of 79 variables on cholesterol, phospholipid, triglyceride and Apo protein content. Several of these variables were correlated. Especially high correlations were observed between the phospholipid or Apo-B100 content and the cholesterol content (Additional Fig. 1). As also the associations between the phospholipid or Apo-B100 content with all bone-related outcome variables were similar to that of cholesterol content (Additional Fig. 2), the former were not analyzed any further.

### Statistical analysis

^1^H-NMR measurements were performed in all SHIP-TREND participants with enough biomaterial and successfully completed in 4,340 individuals. To gain a comprehensive understanding of the lipoprotein data and to identify relations between the variables, Pearson correlation was employed. All following analyses were restricted to the study population described above (*n* = 2,471).

General characteristics of the study population were expressed as medians with 25th and 75th quartiles (continuous data) or as proportions (nominal data). Associations between the BTMs (exposure, log-transformed) and the stiffness index (outcome, log-transformed) were examined in unadjusted linear regression models.

Associations between the lipoprotein data (exposure) and the BTMs or stiffness index (outcome) were investigated using linear regression models. As the three outcome variables, but especially P1NP and CTX, were not normally distributed, they were log-transformed before being entered in the models. To ensure comparability of the regression coefficients between the models, we scaled the exposure variables by dividing them by their standard deviation. The effects obtained from all models thus represent a one standard deviation increase in the exposure on the outcome. The selection of covariates followed a literature-based approach. Next to sex and age, we considered waist circumference to be a significant determinant of exposure and outcome, as well as the lifestyle-related variable physical inactivity. Also diabetes mellitus and the hsCRP concentration, as proxy for inflammatory processes, were included as covariates in all models. Finally, to reduce the variation introduced by possible differences between the instruments, all models were adjusted for the device of the ^1^H-NMR measurement. Sex differences and age- or menopause specific changes in lipoprotein and bone metabolism are well known [17, 18]. Effect modification by sex was tested and observed (interaction terms *p* < 0.05) in 111 out of 237 (79 lipoprotein variables x 3 outcome variables) models. Effect modification by menopausal status was observed in 68 out of 237 models in women and effect modification by an age cut-off of 60 years in 158 out of 237 models in men. Therefore, all of the above analyses were performed separately in (1) men younger than 60 years of age, (2) men aged 60 years or older, (3) premenopausal women and (4) postmenopausal women. In these separate data sets the adjustment for sex was omitted, reducing the list of covariates to age, waist circumference, physical inactivity, diabetes mellitus, the hsCRP concentration and the device of the ^1^H-NMR measurement.

Finally, for total cholesterol and total triglycerides we examined whether non-linear models (restricted cubic splines with three knots at the 5th, 50th and 95th percentile) had a better fit than linear models.

The bone resorption marker CTX underlies a circadian rhythm and changes postprandially [19]. Therefore, in a sensitivity analysis, we recalculated all models with P1NP or CTX as outcome after exclusion of non-fasting participants or participants with blood samping after 10.00 a.m.

To account for multiple testing, we adjusted the *p*-values by controlling the false discovery rate (FDR) at 5% using the Benjamini-Hochberg procedure. All statistical analyses were performed with SAS 9.4 (SAS Institute Inc., Cary, NC, USA).

## Results

General characteristics of the 1,348 men stratified by age in younger and older than 60 years and of the 1,123 women stratified by menopausal status are given in Table 1.

**Table 1:** Characteristics of the study population. Data are median (1st -3rd quartile) or proportions. *^1^H-NMR measurement. CTX, carboxy-terminal telopeptide of type I collagen; HDL-cholesterol; high-density lipoprotein cholesterol; hsCRP, high-sensitivity C-reactive protein; LDL-cholesterol, low-density lipoprotein cholesterol; P1NP, intact amino-terminal propeptide of type I procollagen

Characteristics	Men < 60 years (*n* = 1,019)	Men ≥ 60 years (*n* = 329)	Premenopausal women (*n* = 508)	Postmenopausal women (*n* = 615)
Age, years	43.0 (33.0–51.0)	68.0 (64.0–73.0)	39.0 (32.0–44.0)	59.0 (54.0–67.0)
Waist circumference, cm	92.0 (84.8–100.5)	99.2 (92.2–106.1)	77.0 (71.0–85.5)	86.0 (78.3–94.7)
BMI, kg/m²	27.0 (24.6–29.9)	28.5 (25.9–31.1)	24.6 (22.0–28.2)	27.4 (24.5–31.3)
Physical inactivity, %	31.3	25.8	33.7	25.5
Diabetes mellitus, %	4.02	17.9	2.76	9.43
HsCRP, mg/l	0.91 (0.51–1.81)	1.46 (0.76–2.86)	0.92 (0.43–2.07)	1.34 (0.76–2.92)
P1NP, ng/ml	48.7 (37.9–62.4)	38.3 (30.2–49.4)	41.9 (33.0–55.5)	51.9 (40.5–66.9)
CTX, ng/ml	0.31 (0.21–0.44)	0.22 (0.14–0.33)	0.20 (0.13–0.30)	0.31 (0.21–0.44)
Stiffness Index	98 (87–112)	91 (80–104)	96 (86–109)	85 (75–95)
Osteoporotic fracture risk				
low, %	73.8	60.8	77.2	50.1
moderate, %	23.6	31.6	21.5	40.0
high, %	2.65	7.60	1.38	9.92
Total-cholesterol, mmol/l*	5.54 (4.75–6.46)	5.73 (5.03–6.54)	5.31 (4.69–5.99)	6.47 (5.64–7.22)
LDL-cholesterol, mmol/l*	1.27 (0.87–1.96)	1.41 (1.06–1.90)	0.85 (0.65–1.23)	1.25 (0.91–1.69)
HDL-cholesterol, mmol/l*	3.39 (2.80–4.04)	3.61 (3.00–4.20)	3.22 (2.65–3.72)	3.96 (3.35–4.62)
Triglycerides, mmol/l*	1.37 (1.19–1.60)	1.41 (1.22–1.59)	1.65 (1.43–1.90)	1.73 (1.46–2.02)

**Fig. 2:**
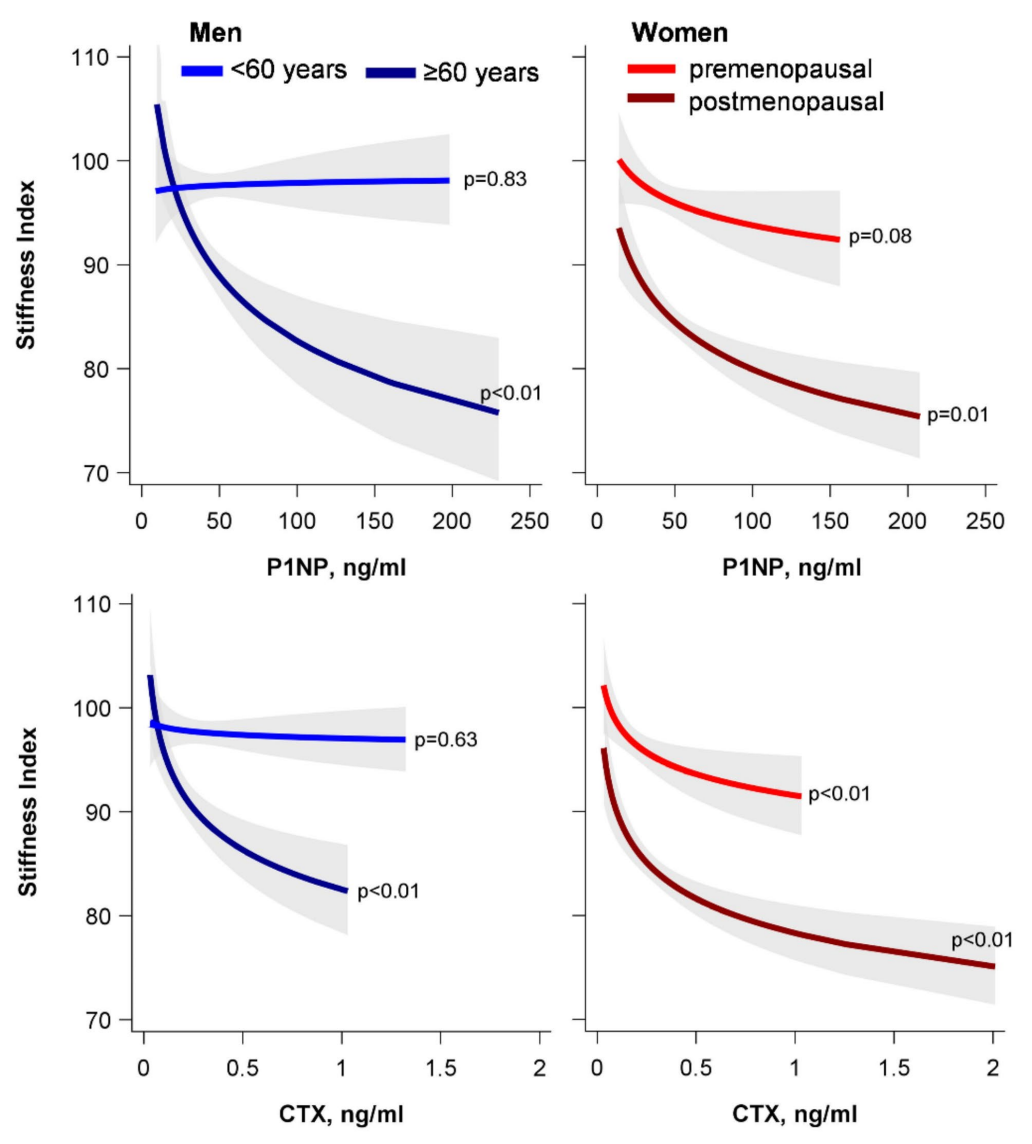
Associations between the bone turnover markers (P1NP and CTX) and the ultrasound-based stiffness index. Results from unadjusted linear regression models in men younger than 60 years (*n* = 1,019), men aged 60 years or older (*n* = 329), premenopausal (*n* = 508) and postmenopausal women (*n* = 615). P1NP, CTX and the stiffness index were log-transformed before being entered in the regression models. The effect estimates were back-transformed for the illustration. CTX, carboxy-terminal telopeptide of type I collagen; P1NP, intact amino-terminal propeptide of type I procollagen

Men aged 60 years or older and postmenopausal women had higher waist circumferences, higher hsCRP concentrations, more often diabetes mellitus, a lower stiffness index and more often a moderate or high osteoporotic fracture risk than younger men and premenopausal women, respectively. P1NP or CTX concentrations were lower in older than in younger men, but higher in post- than in premenopausal women. In addition, the ultrasound-based stiffness index decreased with increasing BTMs in all subgroups except for younger men, in whom, the BTMs and stiffness index were unrelated (Fig. 2).

**Fig. 3:**
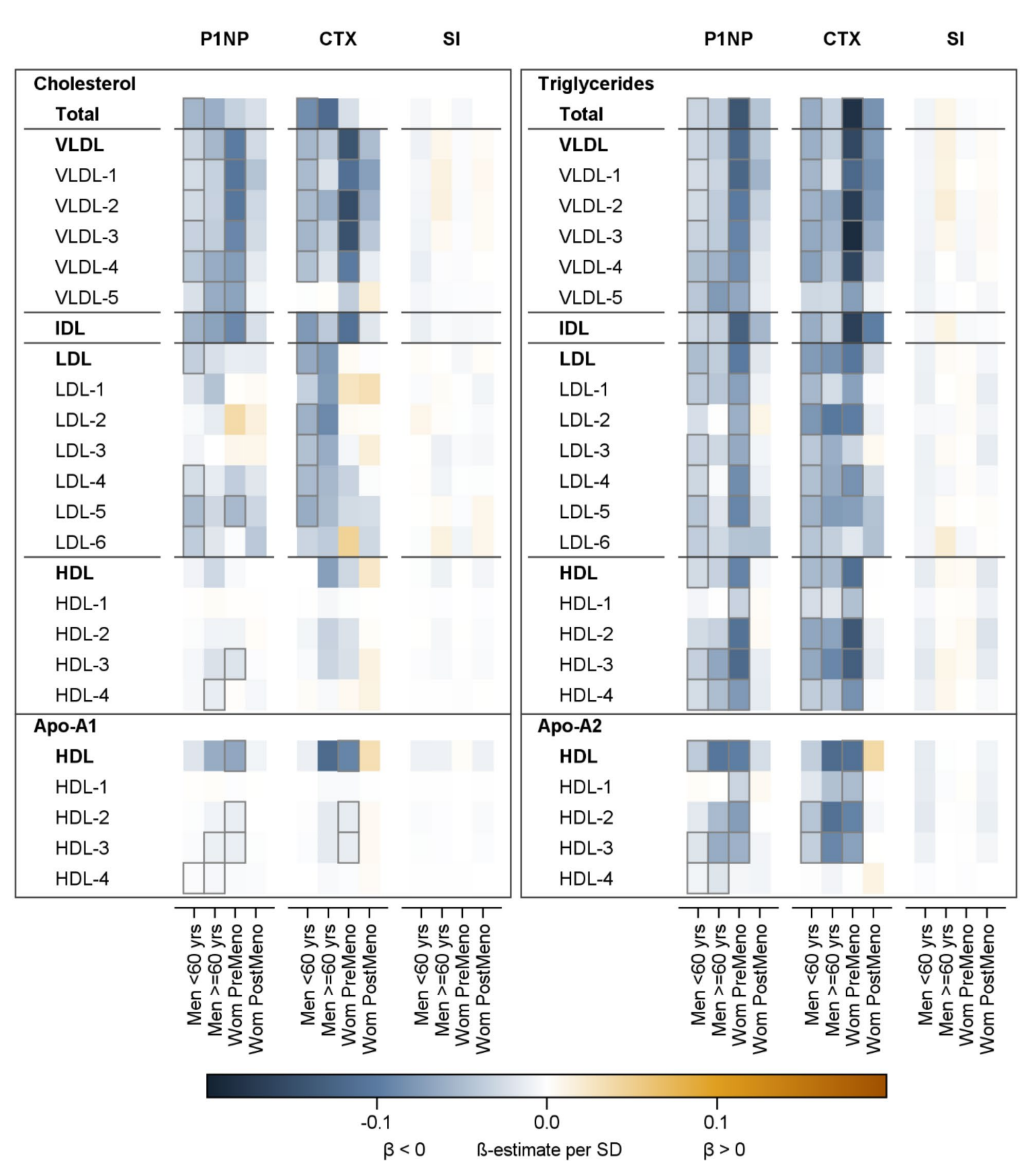
Association between the lipoprotein subclasses and P1NP, CTX and the ultrasound-based stiffness index (SI). The heatmap illustrates the ß-coefficients from linear regression models. Orange and blue shading indicates positive and inverse associations, respectively. Significant associations (FDR-adjusted *p* values < 0.05) are marked with a black box. The effect of a one standard deviation (SD) increase in the exposure on the log-transformed P1NP, CTX and stiffness index is given. The models were calculated separately for men younger than 60 years (*n* = 1,019), men 60 years or older (*n* = 329), premenopausal women (*n* = 508) and postmenopausal women (*n* = 615). All models were adjusted for waist circumference, physical inactivity, diabetes mellitus, high-sensitivity C-reactive protein concentration and device of ^1^H-NMR measurement. CTX, carboxy-terminal telopeptide of type I collagen; FDR, false discovery rate; P1NP, intact amino-terminal propeptide of type I procollagen

The results of all following analyses revealed some remarkable, general findings. First, while several associations between the lipoprotein subclasses and P1NP and CTX concentrations were present, there was no evidence for any association with the ultrasound-based stiffness index. Second, P1NP and CTX concentrations showed similar, inverse associations. Third, there were more associations in younger than in older individuals (Fig. 3).

In detail, our analyses demonstrated very clear and consistent results regarding VLDL and IDL particles. In men younger than 60 years and in premenopausal women, the cholesterol and triglyceride content in total VLDL, VLDL1-4 and IDL particles was inversely associated with P1NP and CTX concentrations and thus with a lower bone turnover. In men aged 60 years or older and in postmenopausal women, effect estimates for these associations were similar or slightly attenuated but only few reached statistical significance.

In LDL particles (total and LDL1-6), the triglyceride content was inversely associated with P1NP and CTX concentrations in younger men and premenopausal women. Associations between the cholesterol content in LDL particles (total and LDL1-6 subclasses) and P1NP and CTX concentrations were restricted to younger men. In men aged 60 years or older and in postmenopausal women, significant associations were absent, yet, effect estimates were quite consistent for the associations between triglycerides or cholesterol content and CTX concentrations.

In HDL particles (total and HDL1-4), inverse association between the triglyceride content and both BTMs were observed in younger individuals of both sexes. Several associations, especially with total HDL, HDL-3 and HDL-4 particles were confirmed in older individuals. Further, the detected associations for the Apo-A2 content in HDL subclasses were clearly comparable with those observed for the triglyceride content. In contrast, the cholesterol content in HDL particles was not consistently associated with either of the BTMs and effects estimates were low. A comparable lack of association was found for the Apo-A1 content.

In the sensitivity analysis, excluding non-fasting participants and participants with blood samping after 10.00 a.m., we examined fewer statistically significant associations between the lipoprotein subclasses and the P1NP or CTX concentrations (Additional Figs. 3 and 4). Especially, effects with CTX in men were attenuated. Yet, the general patterns of the associations remained stable. Thus, triglyceride and cholesterol content in VLDL and IDL particles as well as triglyceride content in LDL and HDL particles and Apo-A2 in HDL particles demonstrated inverse relations with the BTMs.

## Discussion

In this cross-sectional study, we noticed inverse associations between the lipid content of selected lipoproteins and adult bone turnover. An increase in the triglyceride or cholesterol content of VLDL and IDL particles was related to lower P1NP and CTX concentrations, especially in younger individuals. Similar effects were observed for the triglyceride content of LDL and HDL particles and for the Apo-A2 content of HDL particles. These effects were independent of the lipoprotein subclass. Associations between the lipid content of the lipoproteins and the ultrasound-based stiffness index were absent.

Our explorative study showed considerable inverse associations between the lipid content of the examined lipoproteins and both BTMs. Similar associations of P1NP and CTX were expectable, as in non-selected adults, bone formation and resorption processes are coupled [20]. Although with aging, bone resorption outweighs bone formation, a general increase in BTM concentrations is observed in women after menopause [20]. Elevated BTMs in turn, are associated with an increased fracture risk in postmenopausal women [21]. Our data revealed that both BTMs are inversely associated with the triglyceride and the cholesterol content of VLDL and IDL particles. These associations were consistent over the VLDL subclasses, between the sexes, present in younger and older individuals, and therefore of especial interest.

VLDL and IDL are triglyceride-rich particles. They transport triglycerides, but also cholesterol, to peripheral tissues such as muscles or adipose tissue, where they are hydrolyzed to release free fatty acids (FFA) for energy production or storage in intracellular lipid droplets [2, 3]. The observed inverse associations between the triglyceride and cholesterol content in VLDL and IDL particles with P1NP and CTX, point to a stable, possibly protective effect of the triglyceride-rich particles on bone turnover. Bone remodeling, which is crucial to maintain bone strength, is an energy-consuming process and an adequate supply with glucose and FFAs is essential for skeletal health [22]. Osteoblasts use fatty acid oxidation for energy production. Blocking this process results in a low-bone-mass phenotype in mice [23]. Further, FFAs indirectly impact on osteoclast development and function by activating nuclear transcription factors, as NFATc1 and NF-κB (reviewed in [24]). Hence, FFAs and triglycerides play an important role in bone homeostasis [25]. In line with this, Dragojevic et al. [26] reported that bone biopsies from fifty osteoporotic patients vs. fourteen healthy controls not only exhibited lower osteoblastogenesis, but also impaired triglyceride metabolism, characterized by an impaired fatty acid uptake and release from bone cells. Of note, the vast majority of the study participants had normal triglyceride concentrations (< 2.30 mmol/l, 88.1%). Thus, the positive association between triglycerides and bone turnover was largely restricted to the normal range.

Also cholesterol plays an important role in skeletal integrity by exerting multiple direct and indirect effects on bone cells. For example, it is a precursor for the synthesis of steroid hormones like estrogen, testosterone and vitamin D, all of which are critical for bone health. Furthermore, cholesterol represents an essential component of bone cell membranes [22]. Moreover, cholesterol modulates the osteoblastic differentiation of mesenchymal stem cells [27], plays a crucial role in signal transduction during osteoclastogenesis and increases osteoclast viability [28]. Potential protective effects of VLDL-1 particles have been reported from a community-based study, performed in 797 Chinese adults [29], which found that the chance of having osteoporosis decreased with increasing lipid content in VLDL-1 particles, including for example VLDL-1 triglycerides and cholesterol. Another study, restricted to female participants (483 pre- and 118 postmenopausal), reported positive associations of VLDLs with high vs. low BMD in postmenopausal women [30]. In our data, inverse associations between the lipid content of LDL- and HDL-particles and the BTMs were consistent for the triglycerides but largely absent for cholesterol. Only in the subgroup of young male participants, associations of LDL-cholesterol with the BTMs were observed. In the cholesterol-rich LDL- and HDL-particles, the correlation between the triglyceride and the cholesterol content was much lower (Pearson correlation coefficients mainly < 0.5) than in the VLDL and IDL particles (all > 0.7, Additional Fig. 1). It is thus conceivable that the associations with the BTMs observed here are dominated by the triglycerides. However, as functional data is absent, this hypothesis must remain speculative.

A steady and sufficient supply with triglycerides and cholesterol is necessary for bone health. While this is beyond dispute, the detrimental effects of dyslipidemia for bone homeostasis are also clear. Cross-sectional studies suggest that triglycerides are inversely associated with whole body-BMD [31]. In addition, hypercholesterolemia, increased LDL-cholesterol and elevated triglycerides are associated with an increased risk of osteoporosis [32, 33]. A Mendelian randomization study [34] further suggested that statins might positively affect BMD and reduce fracture risk. This effect was attributed to their LDL-lowering effect [34]. Several pathophysiological explanations have been presented that describe how dyslipidemia might affect bone homeostasis. All illustrate the detrimental effects of excessive or impaired lipid metabolism. For example, it has been observed in-vitro that cholesterol-treated mouse osteoblasts demonstrate an impaired proliferation and differentiation [35] and that an atherogenic diet with high cholesterol levels increases the expression of RANKL in mice, which enhances osteoclast differentiation and leads to bone loss [36]. A couple of recent reviews provide more details on the action of lipids on osteoblasts [8, 37], osteoclasts [38] or bone health in general [5, 39]. In addition to the direct effects mentioned above, dyslipidemia exerts several indirect effects on bone health, e.g. by its close relation to obesity. An accumulation of body weight and fat mass is, due to high mechanical loading, an effective stimulus for bone formation [40]. On the other side, obesity causes increased oxidative stress, systemic inflammation, insulin resistance and bone marrow adiposity. Pro-Inflammatory cytokines and adipocytes, as leptin or chemerin, are known to be upregulated in obesity and to inhibit bone physiology [41, 42]. The adipokine chemerin may for example cause a shift in the differentiation of mesenchymal stem cells and promote adipogenesis over osteoblastogenesis [43]. Independent of the specific mechanism, it must be noted that for functional bone remodeling a fine balance of enough but not excess supply with triglycerides and cholesterol is necessary. Our results, combined with the existing literature, suggest a U-shaped association between lipoproteins and bone turnover, with low and high levels adversely affecting bone metabolism. We therefore tested for the presence of non-linear associations between total cholesterol and total triglyceride concentrations and P1NP, CTX or stiffness index. For stiffness index, none of the models indicated a non-linear fit, and for cholesterol only one of the eight remaining tested models indicated a non-linear fit. The results were different for triglycerides (Additional Fig. 5), for which we found reverse J-shaped relations in younger men (P1NP and CTX), older men (CTX) and premenopausal women (P1NP and CTX). In detail, a decrease in triglycerides in the range of 0–2 mmol/l is related to a strong increase in BTMs, whereas in the range above 2 mmol/l BTM concentrations are rather stable. However, our data in this range are sparse and an interpretation is hardly possible. We therefore, cannot finally confirm or refute detrimental effects of elevated triglycerides on bone metabolism.

Despite the consistent effects of the lipids on both BTMs in our study, no associations with the stiffness index were observed. This may have different reasons. The single BTM and lipid measurements examined in our study represent an instantaneous picture of an individual’s bone and lipid metabolism. Yet, alterations of bone substance are subject to slow changes. Thus, effects of an impaired lipid metabolism on bone substance may only become apparent after several years of untreated dyslipidemia, but not in our cross-sectional analyses. Next to lipid metabolism, a multitude of factors affect bone turnover. Indeed, intake of various drugs, such as glucocorticoids, but also the individual medical history and lifestyle-related factors, such as low body weight, all have an impact on BMD [44]. We excluded individuals with intake of glucocorticoids, anti-osteoporotic or lipidlowering medication and adjusted our models for waist circumference, physical inactivity, diabetes mellitus and hsCRP concentration to appropriately consider these effects. But again, the long-term effects of individual medical conditions and lifestyle-related factors or their changes on BMD, could not be assessed in the present cross-sectional analyses. This also fits to our observation of fewer associations in older men and postmenopausal women than in youger men and premenopausal women. Indeed, the observation of more associations in younger than in older individuals was unexpected and explanations must remain speculative. In younger age bone turnover is more stable [20] and general health better than in older age. In this period of life, single risk factors may exert a more pronounced effect than in later life, when multiple risk factors simultaneously impact on bone metabolism. It is therefore possible that the effects of alterations in lipid metabolism on bone turnover are simply more visible here. Young and middle-age men and women should thus be aware of their bone health, including sufficient supply with triglycerides, to prevent disturbances in bone metabolism and deterioration of bone substance. With menopause, BTMs and the osteoporotic fracture risk strongly increase in women [44] and metabolic derangements accumulate. The assumed positive effects of a sufficient supply of bone cells with triglyceride or cholesterol may be exceeded by the detrimental effects of obesity. In fact, the average study participant was overweight, with one quarter of them being classified as obese.

Next to the absence of associations between the lipoprotein subclasses and the ultrasound-based bone stiffness index, our data did not show differential effects of the lipoprotein subclasses on bone turnover. Lipoprotein subclasses possess a distinct cardiovascular risk. For example, the atherogenity of small and dense LDL-particles is higher than that of large, less dense particles [9, 12, 45]. However, the lipoprotein subclasses had similar effects on bone turnover. This suggests that differentiating lipoprotein particles based on their density and size does not provide additional insights into its relation with bone turnover. Future studies might therefore examine other aspects of the relationship between lipid and bone metabolism, e.g. the effects of certain fatty acids. Long-chain polyunsaturated fatty acids, especially omega-3 fatty acids, are anti-inflammatory and thought to be beneficial for bone health. Also, saturated fatty acids and, most importantly, the right balance of the different fatty acid types are needed to maintain healthy bones (reviewed in [46]).

The work presented here stands out due to the large number of participants from the general population with ^1^H-NMR-based lipoprotein quantification. Indeed, our participants cover a large age range, which allowed us to perform analyses stratified by age or menopausal status. Furthermore, the intensive SHIP examinations with highly standardised procedures and validated interviews assured a high data quality and allowed us to carefully select our study population and to adjust our models for interfering covariates. Another strength of our study was the possibility to assess bone turnover and the ultrasound-based stiffness index simultaneously to obtain a detailed picture of the participants’ bone health.

Beside these strengths, our study has at least three limitations. First, our analyses were based on cross-sectional data, which prohibits drawing causative conclusions. Second, BMD measurements based on ionizing radiation represent the gold standard method for osteoporosis diagnostic, but were unavailable in the present population-based study for ethical reasons. However, the present ultrasound-based results provide a comparable fracture risk prediction [47]. Finally, while the SHIP-TREND sample is population-based we excluded selected study participants due to interfering medication intake or medical condition. Our results may therefore not apply to individuals with these criteria. Moreover, our study was performed exclusively in Caucasian participants and may not be directly transferrable to other ethnicities.

## Conclusions

Our results regarding the effects of triglycerides on bone formation and resorption markers are highly consistent. All observed associations in all lipoprotein subclasses had the same direction, arguing for a general protective effect of lipids on bone health at least in the normal range, but against a relevant gain of information by assessing the lipoprotein subclasses. Further, the presented effects on bone turnover did not translate into effects on the ultrasound-based bone stiffness. Nevertheless, our study highlights the close relation between lipid and bone metabolism in younger and older individuals from the general population and the relevance of early awareness of bone-protective behaviour.

## Electronic supplementary material

Below is the link to the electronic supplementary material.

Supplementary Material 1

## Data Availability

Restrictions apply to the availability of data generated or analyzed during this study to preserve patient confidentiality or because they were used under license. Data can be applied for following a standardized procedure: http://www2.medizin.uni-greifswald.de/cm/fv/ship/daten-beantragen/.

## Abbreviations

BMD: Bone mineral density
BTM: Bone turnover markers
CTX: Carboxy-terminal telopeptide of type I collagen
FDR: False discovery rate
FFA: Free fatty acids
^1^H-NMR: Proton nuclear magnetic resonance spectroscopy
HDL: High density lipoprotein
IDL: Intermediate density lipoprotein
LDL: Low density lipoprotein
P1NP: Intact amino-terminal propeptide of type I procollagen
SD: Standard deviation
SHIP-TREND: Study of Health in Pomerania-TREND
VLDL: Very low density lipoprotein
