# Supplementary material for: Bone turnover: the role of lipoproteins in a population-based study
Source: Lipids Health Dis. 2024 Sep 19;23:302. doi: 10.1186/s12944-024-02290-y (PMC11411916; doi:10.1186/s12944-024-02290-y)
Supplement: Supplementary file 1 — Supplementary Material 1 [file 12944_2024_2290_MOESM1_ESM.docx]

**Additional Material**

**Additional Methods**

## **Interview, physical examination and laboratory methods**

The SHIP-TREND program offered a broad range of medical examinations to the study participants. These included, for example, a computer-assisted personal interview on lifestyle and medical history, a quantitative ultrasound measurement at the heel and blood sampling [1, 2].

During the personal interview, participants were asked for lifestyle-related factors, medical history and presence of chronic diseases. All individuals reporting either liver cirrhosis, hepatitis or fatty liver disease were classified as suffering from liver disease. Participants reporting either a physician’s diagnosis of diabetes mellitus, intake of antidiabetic medication, who had a HbA1c ≥ 6.5 % or a glucose concentration ≥ 11.1 mmol/l were defined as having diabetes mellitus. All women aged 60 years or older and all women between 40 and 60 years of age without self-reported regular menstrual cycling were considered postmenopausal, all remaining women as premenopausal. Intake of medication was recorded and classified using the anatomical therapeutic chemical classification system (ATC). Glucocorticoids for systemic use were defined as ATC-code H02AB and H02BX. Antiosteoporotic drugs were defined as bisphosphonates (ATC-code M05BA and M05BB), selective estrogen receptor modulators (ATC-code G03XC) and parathyroid hormone (ATC-code H05AA). Further drugs that impact on bone metabolism and represented exclusion criteria were antiepileptics (ATC-code N03), aromatase inhibitors (ATC-code N02BG) and antidepressants (ATC-code N06A). Antidiabetic medication was classified as ATC-code A10, lipidlowering medication as ATC-code C10, intake of oral contraceptives as ATC-code G03A and intake of menopausal hormone therapy as ATC-code G03C, G03D and G03F. Waist circumference was measured during the physical examination midway between the lower rib margin and the iliac crest in the horizontal plane using an inelastic tape with the subject standing comfortably with weight distributed evenly on both feet.

Venous blood samples were taken in the mornings from the cubital vein of participants in the supine position. Information on fasting status and the exact blood sampling time were documented. Serum and plasma samples were stored at -80 °C in the Integrated Research Biobank (Liconic, Lichtenstein) of the University Medicine Greifswald and used in accordance with its regulations [3]. Serum intact amino-terminal propeptide of type 1 procollagen (P1NP) and C-terminal telopeptides of type 1 collagen (CTX) concentrations were determined by automated chemiluminescent immunoassays on the IDS‑iSYS Multi-Discipline Automated Analyser (Immunodiagnostic Systems Limited, Frankfurt am Main, Germany). Serum high-sensitivity C-reactive protein (hsCRP) concentrations were measured by nephelometry on the Dimension VISTA (Siemens Healthcare Diagnostics, Eschborn, Germany). Serum creatinine concentrations were determined with a modified kinetic Jaffé method. The estimated glomerular filtration rate (eGFR) was calculated according to the four-variable Modification of Diet in Renal Disease formula [4]. Renal insufficiency was defined as eGFR < 30 ml/min/1.73m².

**Lipoprotein quantification**

After thawing, 250 μl of plasma were mixed with 250 μl of phosphate buffer [prepared with D2O and contained sodium 3-trimethylsilyl-(2,2,3,3-D4)-1-propionate (TSP) as reference, (pH 7.4)]. Spectra were recorded on one of three Bruker AVANCE-II 600 NMR spectrometer operated by TOPSPIN 3.2 software (both Bruker Biospin, Rheinstetten, Germany), equipped with 5-mm z-gradient probe (Bruker Biospin, Rheinstetten, Germany) and automated tuning and matching (ATMA) unit (Bruker Biospin, Rheinstetten, Germany). Specimens were automatically delivered to the spectrometer via SampleJet (Bruker Biospin, Rheinstetten, Germany) into standard 5 mm NMR tubes. The acquisition temperature was set to 310°K. A standard one-dimensional ^1^H-NMR pulse sequence with suppression of the water peak (NOESYPREAST) was used. The sequence has the form –RD-gz,1-90°-t-90°-tm-gz,2-ACQ, where RD is the relaxation delay (4 s) t is a short delay (~3 µs), 90° represents the 90° RF hard pulse, tm is the mixing time (10 ms), gz,1 and gz,2 are the magnetic field z gradients both applied for 1 ms and ACQ is the acquisition period (2.7 s) collecting 98304 data points at a sweep width of 30 ppm. The receiver gain is set at 90.5 for all experiments. For pre-processing, a line broadening of 0.3 Hz, a zero filling to produce 128 k data points and a first-order phase correction of 0.0 was applied. Spectral processing included zerofilling, linebroadening, Fourier transform and referencing of the chemical shift and determination of the spectral intensity per 1 mmol protons for quantitative referencing. Chemical shifts of plasma spectra were referenced to the CH3-group signal of alanine adjusting it to 1.48 ppm. Spectra were segmented into N = 450 consecutive integrated spectral regions (buckets) of fixed width covering the region from 0.3 ppm to 1.4 ppm. Two subregions, i.e. 1.31-1.35 ppm and 1.16-1.21 ppm where excluded from binning, in order to avoid unwanted influences coming from lactate and ethanol CH3-group signals in later application of the method in typical population cohorts. Finally, the spectra were submitted to data analysis for lipoprotein subclass and apolipoprotein analysis B.I.LISA^TM^ (Bruker BioSpin GmbH Germany). Based on replicates of a pooled plasma sample, which were measured during the year 2023 on two different 600 MHz NMR spectrometer, the coefficients of variation (CV) for total cholesterol, LDL-cholesterol, HDL-cholesterol and triglycerides varied between 2.85%- 4.69%, 3.77% - 6.13%, 3.20% - 5.43% and 4.85% – 5.20%, respectively.

Total cholesterol, low-density lipoprotein (LDL)-cholesterol, high-density lipoprotein (HDL)-cholesterol and triglycerides (TG) measured by ^1^H-NMR were compared to standard laboratory measurements (Dimension Vista 1500, Siemens Healthcare Diagnostics, Eschborn, Germany) using Passing-Bablok regression and Pearson correlation in 4,340 SHIP-TREND participants. The agreement between the methods was good for all four measurands with Pearson correlation coefficients between 0.90 and 0.97 (data not shown). Further, high correlation coefficients were observed between several of the ^1^H-NMR variables (Additional Figure 1). Specifically, the phospholipid and Apo-B100 content were highly correlated with the cholesterol content in all of the examined particles. The associations between phospholipid and Apo-B100 content with all bone-related outcome variables were similar to that of cholesterol content (Additional Figure 2). Therefore, they were not analyzed any further.

**References:**

1. Volzke, H., et al., *Cohort profile: the study of health in Pomerania.* Int J Epidemiol, 2010. **40**(2): p. 294-307.

2. Volzke, H., et al., *Cohort Profile Update: The Study of Health in Pomerania (SHIP).* Int J Epidemiol, 2022. **51**(6): p. e372-e383.

3. Winter, T., et al., *The Integrated Research Biobank of the University Medicine Greifswald.* Open Journal of Bioresources, 2020. **7**(1).

4. Levey, A.S., et al., *A simplified equation to predict glomerular filtration rate from serum creatinine [abstract].* J Am Soc Nephrol, 2000. **11**(A0828).

**Additional Figures**


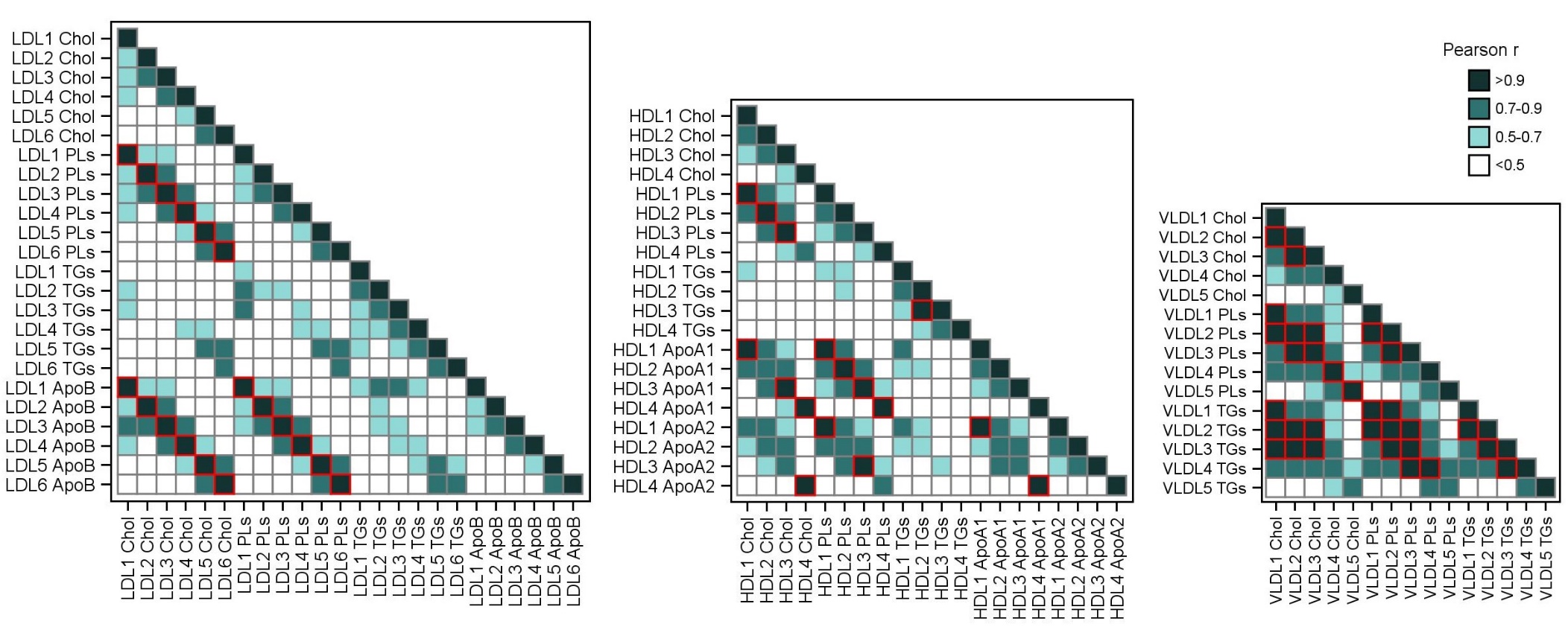


**Additional Figure 1.** Correlation matrices showing within-particle Pearson correlations.

Apo, apolipoprotein; Chol, cholesterol; HDL, high-density lipoprotein; LDL, low-density lipoprotein; PLs, phospholipids; TGs, triglycerides; VLDL, very low-density lipoprotein. ApoB is short for ApoB100 lipoprotein particles.


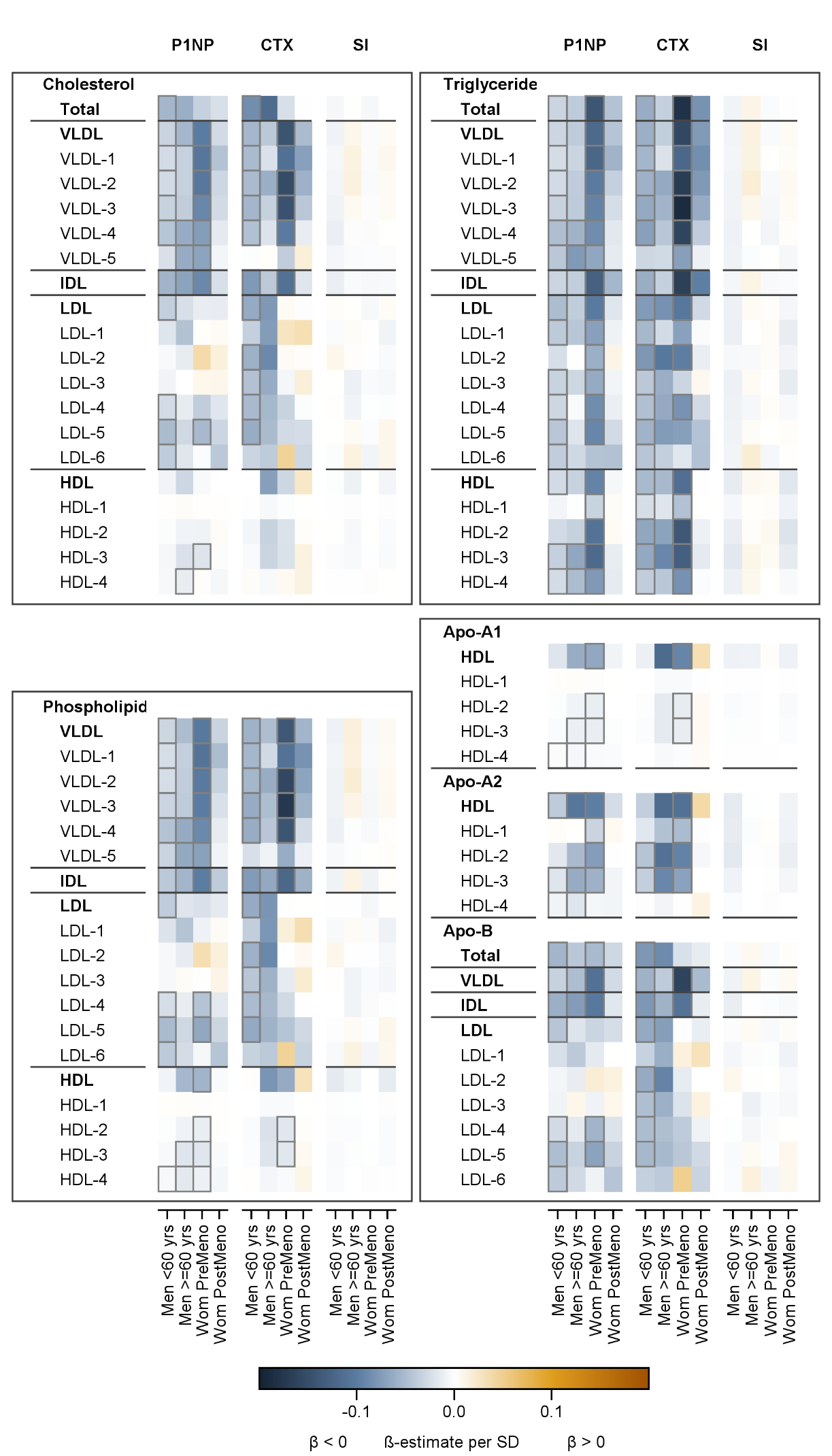


**Additional Figure 2.** Association between the lipoprotein subclasses and the bone turnover markers P1NP and CTX and the ultrasound-based stiffness index (SI) including phospholipids and Apo-B100. The heatmap illustrates the ß-coefficients from linear regression models. Orange and blue shading indicates positive and inverse associations, respectively. Significant associations (FDR-adjusted p values < 0.05) are marked with a black box. The effect of a one standard deviation (SD) increase in the exposure on the log-transformed P1NP, CTX and stiffness index is given. The models were calculated separately for men younger than 60 years (n=1,019), men 60 years or older (n=329), premenopausal women (n=508) and postmenopausal women (n=615). All models were adjusted for waist circumference, physical inactivity, diabetes mellitus, high-sensitivity C-reactive protein concentration and device of ^1^H-NMR measurement.

CTX, carboxy-terminal telopeptide of type I collagen; FDR, false discovery rate; P1NP, intact amino-terminal propeptide of type I procollagen


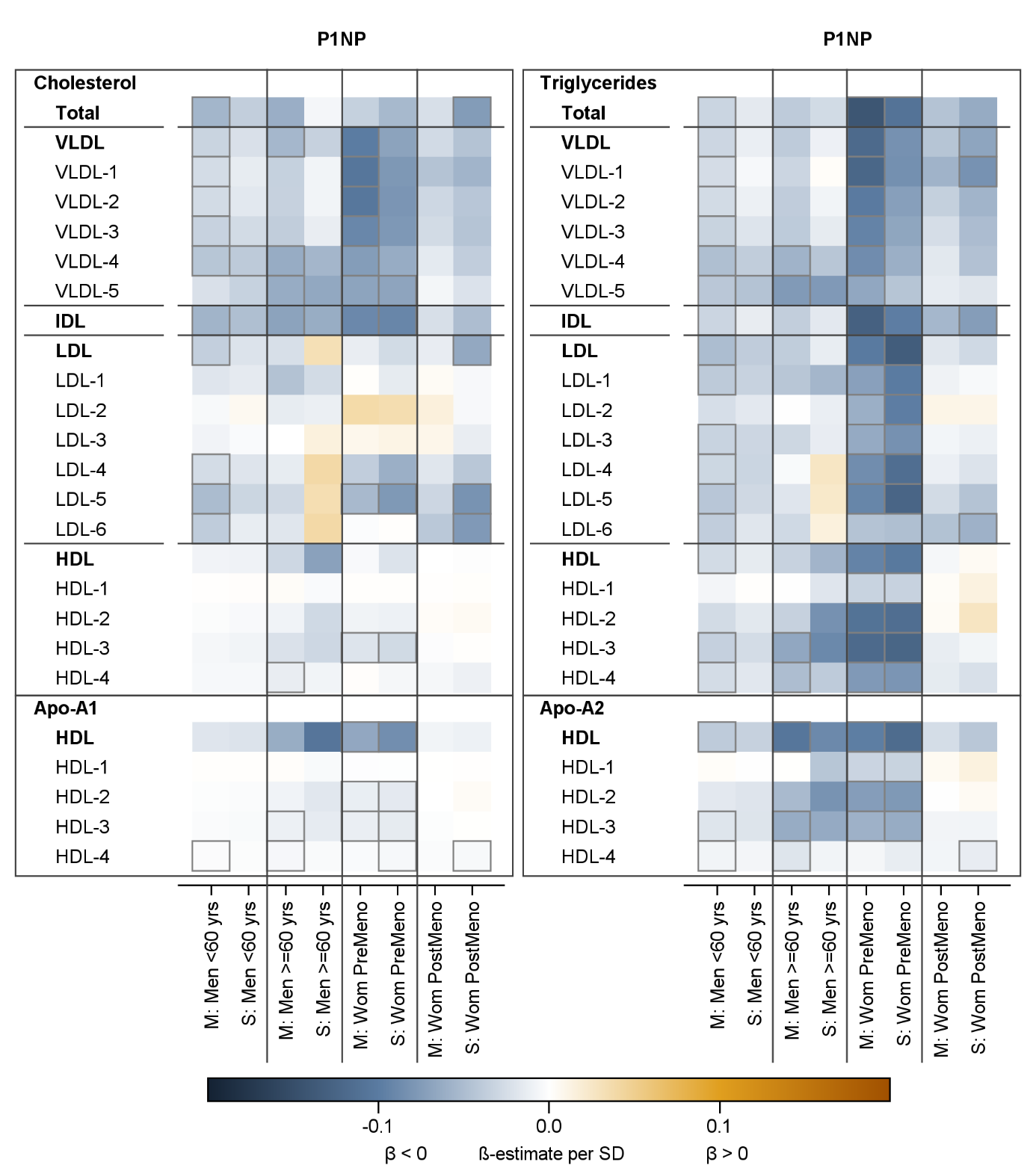


**Additional Figure 3.** Association between the lipoprotein subclasses and the bone turnover marker P1NP in the main (M) and sensitivity analysis (S). For the sensitivity analyses all individuals with blood sampling after 10.00 a.m. and all non-fasting individuals were excluded from the study population. The heatmap illustrates the ß-coefficients from linear regression models. Orange and blue shading indicates positive and inverse associations, respectively. Significant associations (FDR-adjusted p values < 0.05) are marked with a black box. The effect of a one standard deviation (SD) increase in the exposure on the log-transformed P1NP concentration is given. The models were calculated separately for men younger than 60 years (M: n=1,019, S: n=600), men 60 years or older (M: n=329, S: n=165), premenopausal women (M: n=508, S: n=317) and postmenopausal women (M: n=615, S: n=353). All models were adjusted for waist circumference, physical inactivity, diabetes mellitus, high-sensitivity C-reactive protein concentration and device of ^1^H-NMR measurement.

FDR, false discovery rate; P1NP, intact amino-terminal propeptide of type I procollagen


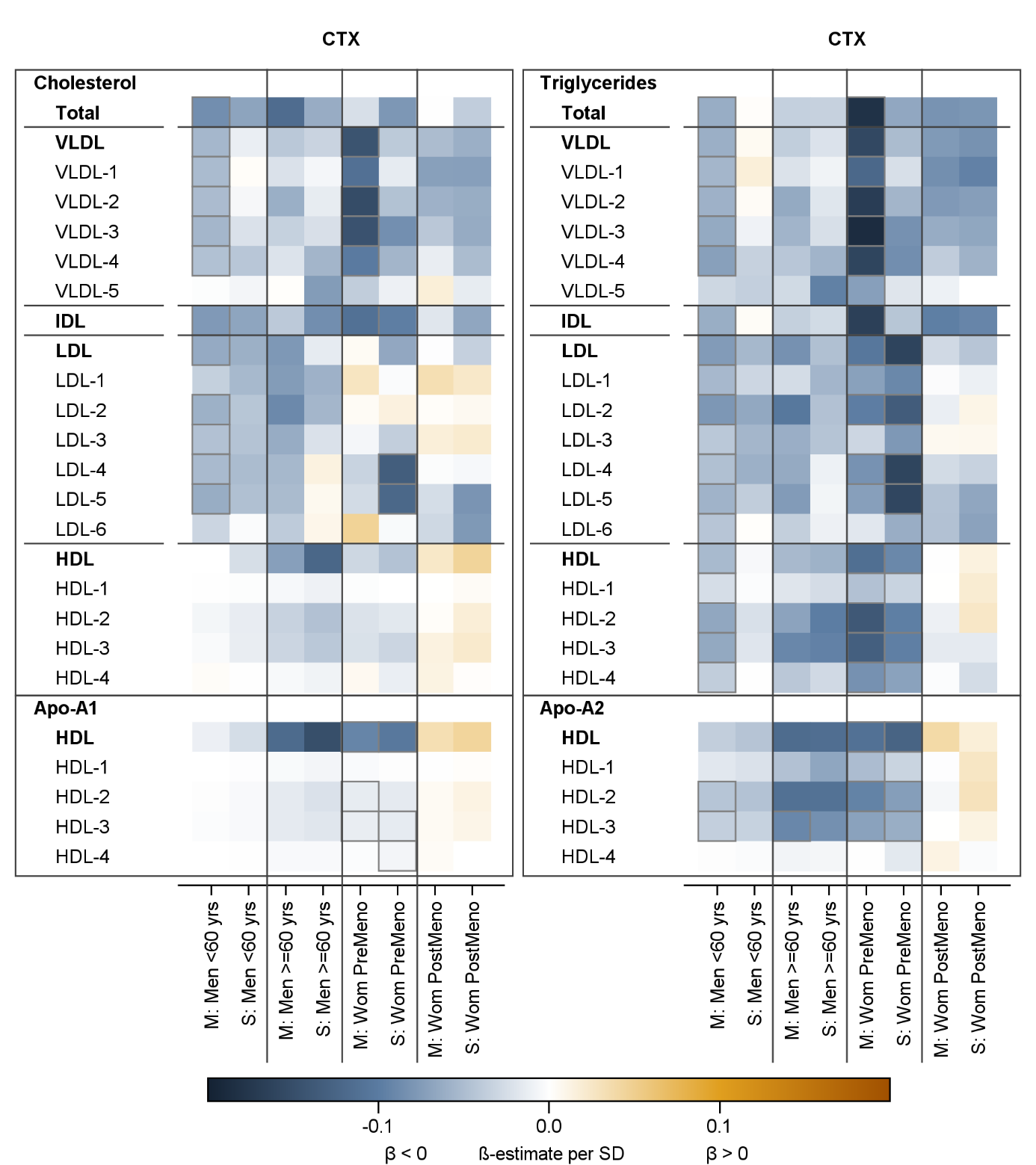


**Additional Figure 4.** Association between the lipoprotein subclasses and the bone turnover marker CTX in the main (M) and sensitivity analysis (S). For the sensitivity analyses all individuals with blood sampling after 10.00 a.m. and all non-fasting individuals were excluded from the study population. The heatmap illustrates the ß-coefficients from linear regression models. Orange and blue shading indicates positive and inverse associations, respectively. Significant associations FDR-adjusted p values < 0.05) are marked with a black box. The effect of a one standard deviation (SD) increase in the exposure on the log-transformed CTX concentration is given. The models were calculated separately for men younger than 60 years (M: n=1,019, S: n=600), men 60 years or older (M: n=329, S: n=165), premenopausal women (M: n=508, S: n=317) and postmenopausal women (M: n=615, S: n=353). All models were adjusted for waist circumference, physical inactivity, diabetes mellitus, high-sensitivity C-reactive protein concentration and device of ^1^H-NMR measurement.

CTX, carboxy-terminal telopeptide of type I collagen; FDR, false discovery rate


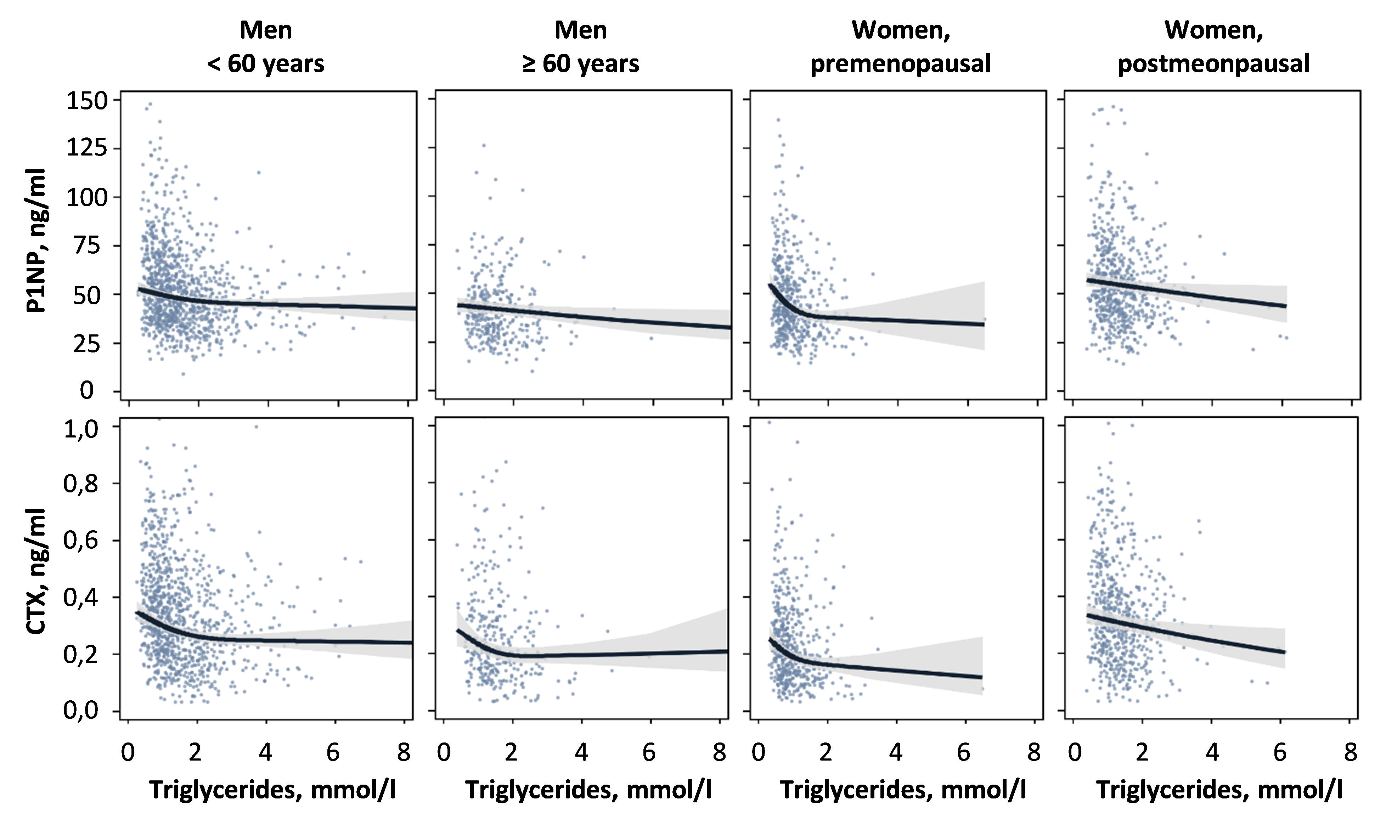


**Additional Figure 5.** Associations between the total triglyceride concentration and P1NP and CTX concentration in men younger than 60 years (n=1,019), men 60 years or older (n=329), premenopausal women (n=508) and postmenopausal women (n=615). Individual values (grey dots) and regression lines (solid black line) with 95% confidence interval from the fully adjusted models are illustrated for an average individual. Non-linear modelling with restricted cubic splines with three knots (5^th^, 50^th^, 95^th^ percentile) was found to have a better fit (indicated by the likelihood ratio test) than linear modelling in younger men (P1NP and CTX), older men (CTX) and premenopausal women (P1NP and CTX).

CTX, carboxy-terminal telopeptide of type I collagen; P1NP, intact amino-terminal propeptide of type I procollagen
